# The Complicated and Confusing Ecology of *Microcystis* Blooms

**DOI:** 10.1128/mBio.00529-20

**Published:** 2020-06-30

**Authors:** Steven W. Wilhelm, George S. Bullerjahn, R. Michael L. McKay

**Affiliations:** a Department of Microbiology, University of Tennessee, Knoxville, Tennessee, USA; b Department of Biological Sciences, Bowling Green State University, Bowling Green, Ohio, USA; c Great Lakes Institute for Environmental Research, University of Windsor, Windsor, Ontario, Canada; University of Georgia

**Keywords:** competition, cyanobacteria, harmful algal blooms, nutrient cycling

## Abstract

Blooms of the toxin-producing cyanobacterium *Microcystis* are increasing globally, leading to the loss of ecosystem services, threats to human health, as well as the deaths of pets and husbandry animals. While nutrient availability is a well-known driver of algal biomass, the factors controlling “who” is present in fresh waters are more complicated. *Microcystis* possesses multiple strategies to adapt to temperature, light, changes in nutrient chemistry, herbivory, and parasitism that provide a selective advantage over its competitors. Moreover, its ability to alter ecosystem pH provides it a further advantage that helps exclude many of its planktonic competitors. While decades of nutrient monitoring have provided us with the tools to predict the accumulation of phytoplankton biomass, here, we point to factors on the horizon that may inform us why *Microcystis* is presently the dominant bloom former in freshwaters around the world.

## Abstract (precis)

Blooms of the toxin-producing cyanobacterium *Microcystis* are increasing globally, leading to the loss of ecosystem services, threats to human health, as well as the deaths of pets and husbandry animals. While nutrient availability is a well-known driver of algal biomass, the factors controlling “who” is present in fresh waters are more complicated. *Microcystis* possesses multiple strategies to adapt to temperature, light, changes in nutrient chemistry, herbivory, and parasitism that provide a selective advantage over its competitors.

## PERSPECTIVE

**FIG 1:**
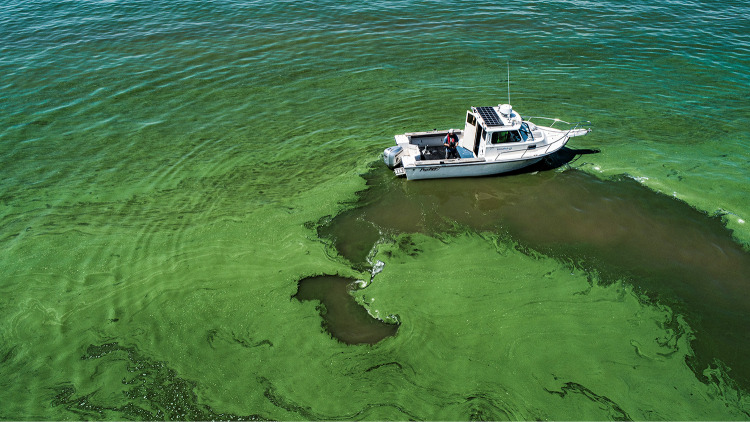
Massive *Microcystis* bloom (3 August 2019) near the mouth of the Maumee River (Ohio) is typical of recent events. (Used with permission from David J. Ruck/Great Lakes Outreach Media).

**FIG 2:**
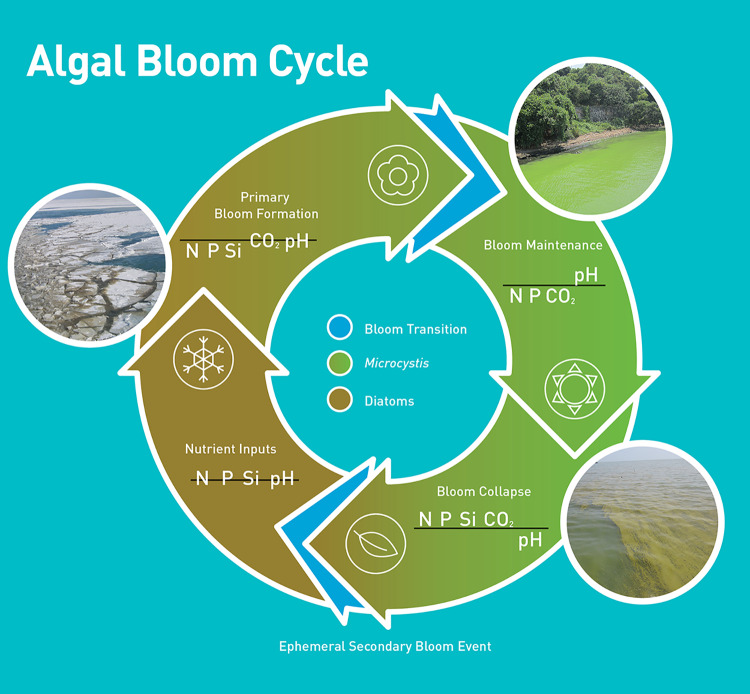
The seasonal cycle of a cyanobacterial bloom in a large dimictic lake. The availability of nutrients (N, P, and Si), dissolved CO_2_, and pH conditions are suggested by the position of acronyms above (high nutrient concentrations or high pH) or below (low nutrients and dissolved CO_2_, lower pH) black marker lines. Acronym positions are relative (no scale implied). Bloom formation in many lakes starts as temperatures increase and stores of nutrients from the winter begin to be consumed and are depleted. As nutrients are depleted and blooms form, cyanobacteria like *Microcystis* are able to drive down CO_2_ concentrations using nutrients that may not be accessible to other planktonic phototrophs. This reduces available CO_2_ and increases pH. As temperatures decrease in fall months, dimictic lakes turn over and “reset” the system.

Expansive seasonal blooms of potentially toxic cyanobacteria now occur globally (Fig. 1 and Fig. 2). Across scales, blooms routinely threaten our water resources, compromising access to potable water, human and animal health, and regional socioeconomics (1, 2). While blooms are well documented, scientists still grapple with why blooms of specific freshwater cyanobacteria occur where and when they do. Understanding the causes and constraints on toxic cyanobacterial blooms is complicated by the nature of the interactions that govern their proliferation.

The accumulation of biomass by phytoplankton is a matter of mass balance; phytoplankton fix carbon dioxide in a relationship proportional to available macronutrients nitrogen (N) and phosphorus (P). This process is constrained by physical parameters, including temperature and light. Generally speaking, macronutrients are the currency facilitating carbon fixation, and thus, nutrient availability often limits both the rate of primary production and biomass accumulation (3). Indeed, the roles of P (4) and N (5) as limiting nutrients of freshwater blooms remain hotly debated yet tied to one truth—more nutrients equals larger blooms. Moving forward, the most direct solution to stopping cyanobacterial bloom events is to reduce nutrient loading. However, this comes with costs that often exceed the current political and socioeconomic will. The main causative organism, *Microcystis*, is a single-celled cyanobacterium that can form buoyant colonies. Subsets of the *Microcystis* community contain the genes needed to synthesize the potent hepatotoxin microcystin, a compound originally known as “fast death factor” (6) that has now been detected in surface waters in 79 countries (7). Beyond this, *Microcystis* produces other bioactive secondary metabolites requiring development of additional risk assessment criteria (8); even nontoxic blooms carry ecosystem-disrupting consequences.

Given the above, a salient question remains why certain phytoplankton proliferate in certain places at certain times. Theories now considered classic have explored the proliferation of phytoplankton and constraints on their diversity (9, 10). Yet science is still unable to answer the common question raised by the citizen constituents: “Why do we get *Microcystis* blooms?” Here, we present a discussion on the many factors influencing bloom formation, persistence, and decline and the research efforts required to understand them.

## 

### Bottom-up controls.

For decades, monitoring efforts have focused on nutrient concentrations as a predictor of phytoplankton biomass. Yet static nutrient concentrations in lakes are as much the residual of biological transformations as they are a cause of blooms. Moreover, all algae need N and P to support carbon fixation, although not all algae assimilate all chemical forms of N and P with similar efficiencies. For example, it is well-known that most marine *Prochlorococcus* do not possess nitrate reductase genes allowing for the assimilation of nitrate. A freshwater parallel may be the assimilation of urea, which has been used increasingly in recent decades as an agricultural fertilizer (3, 11). Urea is an effective N source for many organisms, including *Microcystis* (12, 13). Indeed, the ability to use urea as an N source has been touted as one of the advantages that *Microcystis* has over competing plankton.

*Microcystis* bloom events commonly increase surface water pH to well above 9 as the cyanobacterium rapidly consumes available inorganic carbon (14). Under these conditions, the availability of dissolved CO_2_ to phototrophs is negligible, and even bicarbonate concentrations are low. Numerous researchers have noted that cyanobacterial carbonic anhydrase gives *Microcystis* an advantage in the use of bicarbonate as a carbon source; it should be noted that cyanobacteria are also well adapted to high CO_2_ concentrations (14). However, recent work has demonstrated that urea can also serve as a carbon source for *Microcystis* at alkaline pH (15), offering another selective advantage. Moreover, at pH conditions >9.26, ammonium is converted to ammonia that can diffuse from the system in gaseous form, making more stable N species (e.g., urea and nitrate) important and decreasing total water column N. Perhaps even more importantly, the success of *Microcystis* in raising the pH can create conditions unfavorable for other phytoplankton, e.g., the siliceous frustules of diatoms become soluble, and Si is likely incorporated at lower rates under these pH conditions. That said, pH swings due to rampant photosynthesis are diel processes, yielding shifts of up to 0.5 pH units (15). Typically, pH decreases at night due to respiration without coincident CO_2_ uptake, and thus, diatom success may be linked to whether frustule synthesis can occur at night. Given the paucity of diel studies on bloom gene expression (16, 17), whether pH alone can lead to the exclusion of diatoms requires further investigation.

We note this competition is not restricted to diatoms; pH, along with nutrients and temperature, is a potential driver that can promote *Microcystis* success (or lack of) over other algal taxa and bloom-forming cyanobacteria such as *Planktothrix*, *Dolichospermum*, and *Cylindrospermopsis* (18). Little is known about the factors that constrain their interactions and the outcomes, although predation, as well as nutrient and anthropogenic loads, likely play key roles (1, 19) These observations highlight a key point with respect to *Microcystis* populations: how they compete with one type of organism (e.g., diatoms) is likely different from how they compete with another (e.g., other cyanobacteria).

Increasing temperatures provide another condition that favors some cyanobacteria. *Microcystis* populations grow faster at warmer temperatures (20). Yet toxin production by *Microcystis* cells responds opposite to this trend; *Microcystis* strains in culture produce less toxin per cell when grown at warmer temperatures, consistent with field observations, where blooms accumulate less toxins as the season progresses (21). While reduced toxin production has been linked to the loss of microcystin-producing genotypes from populations (22), the mechanisms that could drive a seasonal and specific gene loss (or selection for populations) remain unclear (especially when that gene returns in subsequent years). Other factors, including the possible role of microcystins in offsetting oxidative stress in cells and the effects of lower temperatures increasing excitation pressure/photoinhibition and the production of oxygen radicals, seem more plausible given the high cellular Fe quota driving Fenton chemistry and the presence of photosensitized pigments (23). Regardless of the mechanism, observations point to the confounding issues of growth, temperature, and toxin production; at lower temperatures (∼18°C), populations have slower growth rates, producing lower biomass, yet cells produce more toxins. At warmer temperatures (∼25°C), biomass is higher, but the toxin cellular quota decreases. Thus, a seasonal shift in toxicity occurs as temperatures increase into the late summer months. Yet temperatures across seasons are not linear; daily swings of 1 to 3°C in the surface mixed layer are common (15), and increased episodic weather associated with climate change (24) may cause water temperature fluctuations that could lead to bursts of toxin production (21). These confounding variables also point to a scientific conundrum: to protect public interests, ecosystem stewards must focus on the toxin per volume water (concentration), as that is where causative issues lie. Yet for scientists to elucidate why *Microcystis* makes toxins, they need to be focusing on cell quotas to understand the process.

### Removal processes.

Accumulation of *Microcystis* biomass also depends on removal mechanisms, namely, grazing, parasites, and virus-mediated lysis. Lab studies have demonstrated that *Microcystis* cells are selectively rejected as pseudofeces by filter-feeding mussels in a process that indirectly promotes *Microcystis* growth (25). Moreover, despite *Microcystis*-specific phage occurring at densities that reach 10^5^ ml^−1^, these cyanobacteria proliferate at high cell densities for extended periods (26). Part of their secret may be in the establishment of a lysogenic relationship with phage; in some other prokaryotes, lysogeny imparts homoimmunity to infections by related viruses (27). Yet episodes of viral lysis have been suggested to release intracellular microcystins into the dissolved phase, complicating water treatment protocols (28). The incoming toxin load can be reduced by flocculation of bloom biomass at the water plant intake, whereas dissolved microcystins bypass this step and require more costly chemical treatment(s) (23). Understanding patterns of lytic versus lysogenic infection and factors contributing to lysogen induction will be useful in developing best practices for water utilities. Moreover, research focusing on the extent to which pathogens influence bloom composition and toxicity is required to enable predictive models. Recently, viruses have been shown to play another understudied role: viral infection of competing plankton may provide *Microcystis* with an advantage. In a slight reinterpretation of the “kill-the-winner” model (29), the presence of viruses infecting competing plankton (e.g., diatoms) may provide another selective advantage for *Microcystis* populations (30).

*Microcystis* has numerous advantages over competing plankton in lakes and can both tolerate and exploit conditions of pH, nutrient availability, temperature, and predation that constrain other plankton. Such conditions depend on season and location, so ecosystem managers and researchers must recognize that each factor may contribute to *Microcystis* success in different ways at different sites around the world. Beyond the N versus P debate regarding constraints on ecosystem productivity (3), research must also focus on what competing plankton cannot do or tolerate in working to understand why *Microcystis* has become globally successful. Moving forward, a balance between laboratory work with cyanobacterial isolates, mesocosm manipulations, and fieldwork examining microbial community dynamics will be critical as the effects of competition with other algae, constraints imparted by the co-occurring microbial community (phycosphere), and shifting pressures due to climate change are addressed. Yet for all these complications, integration of data from molecular biology and physiology with remote-sensing of increasingly “smart” lakes (31) will provide a path forward in the protection of our most valuable natural resource: clean, potable water.
